# Cancellation of Bessel beam side lobes for high-contrast light sheet microscopy

**DOI:** 10.1038/s41598-018-35006-1

**Published:** 2018-11-21

**Authors:** Giuseppe Di Domenico, Giancarlo Ruocco, Cristina Colosi, Eugenio DelRe, Giuseppe Antonacci

**Affiliations:** 1 Dipartimento di Fisica, Universita di Roma “La Sapienza”, 00185 Rome, Italy; 2 Center for Life Nano Science@Sapienza, Istituto Italiano di Tecnologia, 00161 Rome, Italy; 3 Institute for Complex System, ISC-CNR, 00185 Rome, Italy; 4 Photonics Research Group, Ghent University – imec, Department of Information Technology, Ghent, Belgium

## Abstract

An ideal illumination for light sheet fluorescence microscopy entails both a localized and a propagation invariant optical field. Bessel beams and Airy beams satisfy these conditions, but their non-diffracting feature comes at the cost of the presence of high-energy side lobes that notably degrade the imaging contrast and induce photobleaching. Here, we demonstrate the use of a light droplet illumination whose side lobes are suppressed by interfering Bessel beams of specific k-vectors. Our droplet illumination readily achieves more than 50% extinction of the light distributed across the Bessel side lobes, providing a more efficient energy localization without loss in transverse resolution. In a standard light sheet fluorescence microscope, we demonstrate a two-fold contrast enhancement imaging micron-scale fluorescent beads. Results pave the way to new opportunities for rapid and deep *in vivo* observations of large-scale biological systems.

## Introduction

One of the main challenges in optical imaging is enabling a high spatiotemporal resolution while providing an efficient light distribution across the samples. Whereas the finite numerical aperture (NA) of lenses imposes a limit on the achievable spatial resolution, the geometry that is used to illuminate and collect light from a sample balances the light distribution efficiency. Standard wide-field microscopes, for example, employ a collimated beam of light to illuminate a sample in either reflection (epi) or transmission mode. This results in a poor contrast due to the amount of out-of-focus light distributed across a sample and collected by the system. In scanning confocal microscopy, this problem has been overcome by the optical sectioning capability enabled by the use of spatial filters (e.g. pinholes) placed at the object conjugate plane whose size matches the one defined by the system point spread function (PSF)^1^. Nevertheless, besides a relatively low temporal resolution, confocal schemes involve extended out-of-focus volumes within the sample to be fully illuminated, in turn potentially inducing sample photodamage.

An efficient method to perform rapid volumetric imaging with minimal sample exposure is given by light sheet microscopy^2–5^. This method involves a dark-field arrangement with two orthogonal objective lenses that separately perform the excitation and the detection of the sample fluorescence signal. In particular, the sample is illuminated with a thin sheet of light and the emitted fluorescence signal is collected along the axis perpendicular (right angle) to the plane of illumination, thus providing a transverse and axial resolution given by the product of the illumination and collection PSFs^6–8^. Light-sheet microscopy is particularly suited for imaging deep in semi-transparent tissues^9–11^ and whole organisms^12^ at high acquisition rates. Moreover, a sub-diffraction spatial resolution of up to 150 nm has been recently demonstrated using structured illumination^13^. Given the high collection efficiency and the improved photon balance within the samples, specimen photobleaching and phototoxicity^14^ are minimized compared to wide-field or confocal microscopy.

A major limitation in light sheet microscopy is given by the rapid divergence of the light sheet illumination when focusing conventional Gaussian beams. In fact, Gaussian beams are subject to diffraction, which limits the achievable field of view and leads to image artefacts across the edges. To overcome this limit, diffraction-free beams, such as Bessel beams^15^ and Airy beams^16^, have seen extensive use in light sheet microscopy^17,18^. In particular, experiments with Bessel beams have shown improvements in the imaging contrast, the field of view and the penetration dept when compared with Gaussian beams^19^, both using linear and nonlinear excitation^20–22^. These beams can generate a non-diverging sheet of light, but this comes at the cost of a considerable amount of background light that spreads out of the object plane. Bessel beams are indeed characterized by a propagation invariant intensity distribution described by a Bessel function of the first kind and zero-th order, which is composed of a central peak of high intensity followed by high-energy side lobes that significantly affect the imaging contrast and increase sample phototoxicity^23^. Attempts to limit the effect of the beam tails, in turn providing an improved optical sectioning in light sheet microscopy have been made using extended focusing^24^, tiling methods^25,26^ or the use of a physical spatial filter provided by galvo mirrors^27^, but these involve a longer data acquisition time due to the scan requirement along the field of view or require complex synchronizations between the camera rolling shutter and the scanning beam^28^. Other attempts to reduce the high-energy side lobes have been made using multiple slits in a 2D arrangement^29^. However, such beams are pseudodiffracting and involve an increase in the beam waist along the propagation axis.

In this Letter, we demonstrate a diffraction-free light droplet illumination that manifests cancellation of the high energy Bessel side lobes, thus providing a more efficient light distribution for light sheet microscopy. Light droplets are recently demonstrated nondiffracting beams of light of finite depth of focus that are obtained interfering multiple co-axial Bessel beams^30^. Miniaturized and superimposed Bessel beams have been further proposed using volume holography^31,32^, have seen use as highly effective optical traps^33^, optical twezeers^34^ and material processing^35,36^. Similarly to these approaches, our method exploits interference of a set of plane waves whose k-vectors lie on two different co-axial cones. In particular, to theoretically study the phenomenon, we perform the Fourier transform of two concentric annular sources whose aspect ratio provides the maximum confinement of light along the main peak while cancelling the unwanted side lobes through a selective destructive interference. In a standard light sheet fluorescence microscope, our droplet illumination is demonstrated to significantly reduce the undesired out-of-focus light contribution with respect to that given by standard Bessel and Airy beams, resulting in an enhanced imaging contrast.

**Figure 1:**
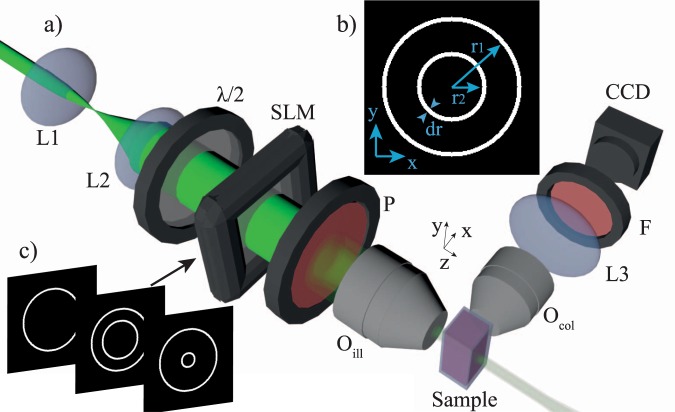
(**a**) Generation of the droplet illumination in a light sheet imaging system. The amplitude of an expanded laser beam was modulated by an SLM and focused by an objective lens (O_ill_) of NA = 0.25. Fluorescence light from a test sample was collected by a second orthogonal objective lens (O_col_) of NA = 0.35 and a tube lens L3. A CCD camera was used to acquire the image by integrating the fluorescence signal emitted during a $\hat{y}$ scan. L_i_ lenses; λ/2 half-wave plate; P linear polarizer; F bandpass filter. (**b**,**c**) To generate the droplet illumination, a set of amplitude binary masks composed of two concentric rings of inner and outer radii, r2 and r1 respectively, and thickness dr were displayed by the SLM.

Figure 1a shows a schematic of the optical setup. The wavefront of an expanded collimated laser beam (*λ* = 532 nm) was modulated by a spatial light modulator (SLM, Holoeye LC2012) that operated in amplitude mode by an appropriate rotation of the input linear polarization, which was set at 45° with respect to the $\hat{y}$ axis using the *λ/*2 waveplate. A linear polarizer was placed after the SLM with the axis parallel to the *y* direction. The amplitude masks displayed by the SLM (Fig. 1b,c) consisted of concentric annular distributions of fixed outer radius (*r*1) of thickness *dr* = 180 μm and varying inner radius (*r*2). An objective lens (Olympus Plan N 10X) of NA = 0.25 was used to perform a Fourier transform of the modulated annular fields and to generate the light droplet illumination. The fluorescent signal from the sample was collected by a second objective lens (Zeiss LD A-Plan 20x Ph1 M27) of NA = 0.35 and a tube lens (Thorlabs TTL200). A bandpass filter (Thorlabs FELH0550) was used to select only the fluorescent signal, which was detected by a CCD camera (Photometrics CoolSNAP HQ2).

To find the ratio *r*_2_*/r*_1_ that provides the maximal energy confinement, we considered the generic electric field describing a zero-order Bessel-Gauss beam^15,37,38^

1 $$\begin{array}{rcl}E(r^{\prime} ,z,\theta ) & = & {E}_{0}\frac{{\omega }_{0}}{\omega (z)}\exp [i((k-\frac{{k}_{r}{(\theta )}^{2}}{2k})z-{\rm{\Phi }}(z))]\\  &  & \times {J}_{0}(\frac{r^{\prime} {k}_{r}(\theta )}{\frac{iz}{{z}_{R}}+1})\exp [(\frac{ik}{2R(z)}-\frac{1}{{\omega }^{2}(z)})(r{^{\prime} }^{2}+\frac{{k}_{r}{(\theta )}^{2}{z}^{2}}{{k}^{2}})],\end{array}$$

where *J*_0_ is a Bessel function of the first kind and zero order, $r^{\prime} =\sqrt{{x}^{2}+{y}^{2}}$ is the radial coordinate, ${k}_{r}(\theta )=k\,sin(\theta )$ and ${k}_{z}(\theta )=k\,cos(\theta )$ are the radial and longitudinal wavevectors respectively (being $k=|{\boldsymbol{k}}|=\sqrt{{k}_{r}{(\theta )}^{2}+{k}_{z}{(\theta )}^{2}}=$ $2\pi /\lambda$ and $\theta ={\rm{atan}}(r/f)$ the angle between the wavevector and the propagation axis), ${z}_{r}=\pi n{\omega }_{0}^{2}/\lambda$ is the Rayleigh length, *ω*_0_ is a measure of the width of the Gaussian term, $\omega (z)={\omega }_{0}\sqrt{1+{(z/{z}_{r})}^{2}}$, $R(z)=z/(1+{z}_{r}^{2})$ is the radius of curvature of the beam’s wavefronts and ${\rm{\Phi }}(z)={\tan }^{-1}(z/{z}_{r})$ is the Gouy phase shift. Thus, the total optical power distributed along the droplet side lobes for given values of *θ*_1_ and *θ*_2_ is given by

2 $$P={\int }_{{r}_{min}^{^{\prime} }}^{\infty }2\pi r^{\prime} {|{E}_{1}(r^{\prime} ,z,{\theta }_{1})+{E}_{2}(r^{\prime} ,z,{\theta }_{2})|}^{2}dr^{\prime} ,$$

where ${r^{\prime} }_{min}$ is the radial distance associated to the first minimum of the intensity pattern.

**Figure 2:**
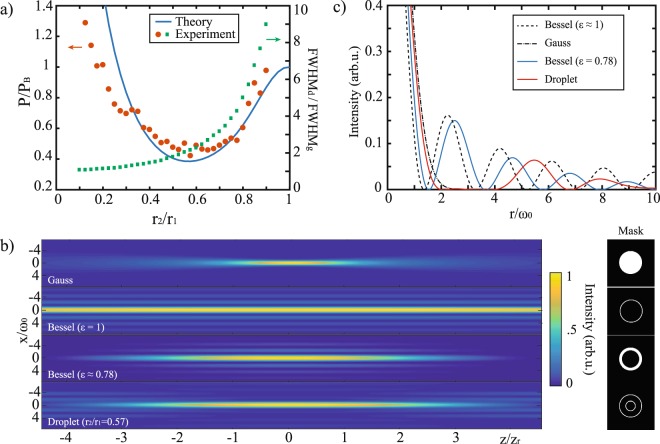
(**a**) Plot of the ratio between the optical power (for *z* = 0) distributed across the droplet side lobes with respect to the Bessel lobe power as a function of the ratio *r*_2_*/r*_1_. The solid line is the theoretical prediction while red dots are experimental data. A plot of the ratio between the axial FWHM of the droplet beam and the Gaussian beam as a function of the ratio *r*_2_*/r*_1_ is reported in the same graph. Top-view (*xz* plane) (**b**) and radial (**c**) intensity profiles of a Gaussian, an ideal Bessel beam ($\epsilon =1$) and a short Bessel beam ($\epsilon =0.78$) of comparable axial FWHM to that of the droplet beam as a function of the number of Rayleigh lengths and for an equal NA. For *r*_2_/*r*_1_ ~ 0.57, the light distributed across the droplet side lobes is about 62% and 48% lower than that of an ideal and a short Bessel beam respectively. Associated beam amplitude masks (inset).

Figure 2a shows the theoretical and measured optical power and the FWHM of the droplet side lobes along the propagation axes. The first quantity has been evaluated as the total amount of power distributed along the droplet beam side lobes and compared with the side lobes power of an equivalent (same peak intensity) Bessel beam measured using the same procedure. The ratios P/P_B_ has been computed for each value of the ratio *r*2*/r*1 both theoretically (we use Eq. 2 with z = 0 both for P and P_B_) and experimentally. The chart also reports the FWHM of the droplet beam along its propagation axis (green squares). The values are normalized with respect to the FWHM of the Gaussian beam with the same waist. A comprehensive plot of the side lobe power along one axial period of the droplet beam can be found in Suppl. Fig. S1. Experimental data was obtained varying the radius *r*2 of the inner annulus on the SLM and detecting the resulting field distribution after Fourier transform. For *r*2*/r*1 close to 1, the droplet beam profile is similar to a standard Bessel beam and the power ratio approaches to 1. On the other hand, when *r*2*/r*1 → 0 this ratio diverges because it corresponds to the superimposition of a Bessel beam with a plane wave. Interestingly, in the region where *r*2*/r*1 is between 0.4 and 0.8, the power in the side lobes is significantly reduced with respect to that of a Bessel beam. The minimum was found to be at *r*2*/r*1 = 0.57, which corresponds to a theoretical 62% reduction of the off-axis light distribution with respect to the ideal Bessel beam. It is also notable that the amount of side lobe light within the range *r*2/*r*1 → (0.4–0.8) remains considerably lower with respect to the standard Bessel beam, allowing flexibility in the choice of the optimal aspect ratio for light sheet illumination. For example, a droplet beam with *r*2*/r*1 ∼ 0.8 that is 5 times longer than a Gaussian beam would give about 40% reduction in the side lobe intensity with respect to the Bessel beam.

Figure 2b compares the theoretical 2D intensity distribution (*xz* plane) given by a standard Gaussian beam, an ideal Bessel beam ($\epsilon$ ∼ 1, where $\epsilon$ = *r*_*out*_*/r*_in_ is a measure of the annulus thickness^39^) and a single droplet beam obtained with the optimal aspect ratio (*r*2*/r*1 = 0.57). The intensity distribution of a short Bessel beam obtained with a thicker annulus ($\epsilon$ ∼ 0.78) of equal axial FWHM to that of the droplet beam is also reported for comparison. The associated radial intensity profiles are plotted in 2c. A full reconstruction of the droplet intensity profiles for *r*2*/r*1 ranging between 0.1 and 1 can be found in Suppl. Video 1. Unlike the diverging Gaussian beam, the Bessel beam manifests an invariant transverse profile along the propagation axis $\hat{z}$. Nevertheless, half of its total energy is distributed along the side lobes. By contrast, our droplet beam maintains an extended depth of focus, ∼2.6 times greater than the one given by the Gaussian beam, but provides a significantly enhanced energy confinement across the central peak without loss in transverse resolution. It needs to be noted that the droplet illumination presents multiple peaks of both equal size and intensity distribution along the z-axis as a consequence of the two co-axial Bessel interference, as previously demonstrated^30^. In this article, we considered the case of a single droplet peak whose axial extend and side lobe intensity can be tuned by changing the ratio between the two annulus radii.

**Figure 3:**
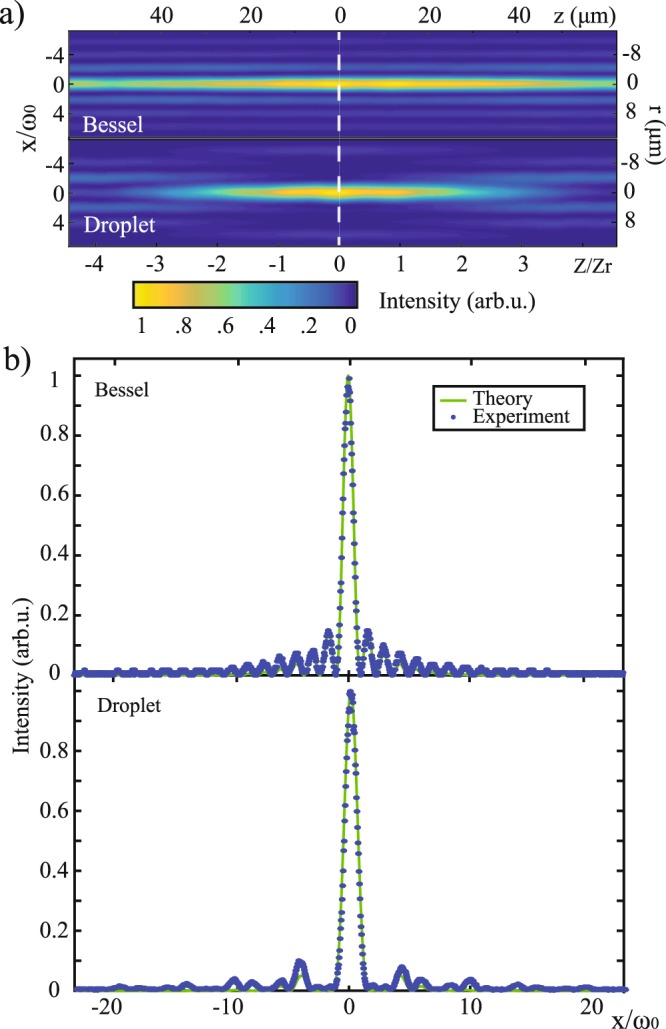
(**a**) Representative Bessel beam obtained with a single ring of radius *r*_1_ (top) and droplet beam with a ratio of *r*2*/r*1 = 0.57 (bottom). (**b**) Measured radial intensity profile of the two beams along the dashed line together with the theoretical prediction (green line). The power distributed across the side lobes of the Bessel beam is suppressed by more than ~50% in the case of the droplet beam.

Figure 3a shows the measured intensity profiles for both a standard Bessel (top) and a droplet beam (bottom). While the Bessel beam was obtained by Fourier transform of a single annular aperture (*r*1 = 1.7 mm diameter and 150 *µ*m width) displayed on the SLM, the illustrated droplet beam resulted from the Fourier transform of two annuli in the case of *r*2*/r*1 = 0.57, where *r*1 was maintained equal and the amplitude normalized to that of the reference Bessel beam. The associated normalized radial intensity profiles for both beams are illustrated in Fig. 3b. Both profiles show a central peak of comparable width. Nevertheless, the droplet beam manifests significantly lower side lobes as a result of the selective destructive interference of two fields of slightly different k-vectors. Integrating the intensity along the radial distribution of the normalized Bessel and droplet beams, we found an overall 57.9% reduction in the side lobes intensity, whilst the intensity of the first side lobes decreased by 88%.

**Figure 4:**
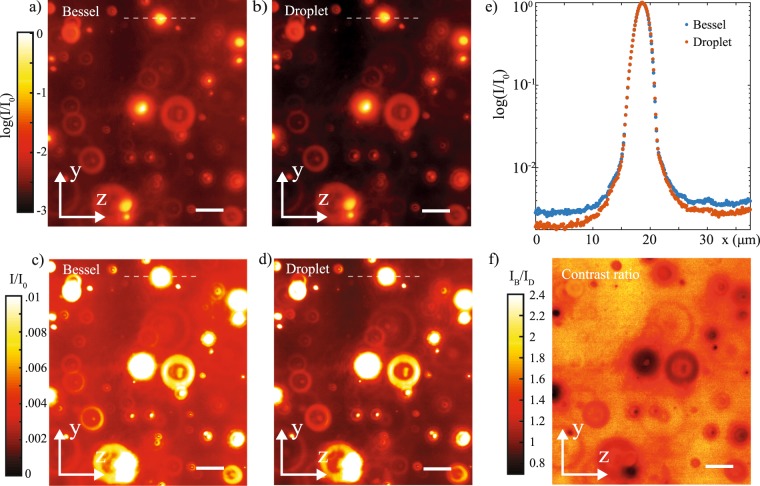
Fluorescence images in both log (**a**,**b**) and linear (**c**,**d**) scales of embedded micron-sized beads obtained with a Bessel beam (**a**,**c**) and a droplet beam illumination (**b**,**d**) in a light sheet microscope. The intensity profiles (**e**) along the dashed line show a two-fold reduction in the background light given by the droplet illumination as a result of the suppression of the out-of-focus side lobes. Scale bar is 10 *µ*m. (**f**) Map the intensity ratio between the images obtained with Bessel and droplet illumination.

The enhanced imaging capability offered by the droplet illumination was demonstrated in a standard light sheet microscope on a fluorescent specimen (Fig. 1a). This was composed of fluorescent spheres with diameters ranging between 5 and 20 *µ*m embedded in a fixed gel mounted on a glass cuvette. Figure 4 shows the images in a log and a linear scale obtained with a standard Bessel beam (Fig. 4a,c) and the droplet illumination (Fig. 4b,d). The images were acquired by scanning the sample along the $\hat{y}$ direction and integrating the emitted fluorescent signal acquired by the CCD camera. The image obtained using a conventional Bessel beam illumination appears with a significant background across the field of view as a consequence of the out-of-focus-light given by the side lobes that is collected by the system. In turn, the associated image obtained using the droplet illumination has a visibly reduced background, the result of the side lobes suppression capability of these beams. Figure 4e illustrates a line profile along a sphere captured across the image field of view, which emphasizes the enhanced contrast provided by the droplet illumination. To give a measure of the background reduction, Fig. 4f illustrates the ratio between the frames obtained with the Bessel and the droplet illuminations. This ratio is close to 1 near the fluorescent spheres and increases elsewhere, indicating an averaged two-fold reduction of the background illumination.

In summary, we demonstrated that the unwanted high-energy side lobes present in common diffractionless optical beams can be effectively suppressed by more than 50% superimposing two co-axial Bessel beams of slightly different k-vectors. The resulting light droplet illumination provides an improved and more efficient energy distribution along the beam profile. While Bessel beams give a theoretically infinite axial extent but present high-energy side lobes, Gaussian beams ideally provide no side lobes but are limited by a shorter depth of focus. In turn, the droplet illumination provides a tradeoff solution consisting of a beam that has a longer depth of focus compared to the Gaussian beam and has significantly lower side lobes than those given by a Bessel beam.

In light sheet fluorescence microscopy, our light droplet illumination results in an increased imaging contrast without affecting the spatiotemporal resolution offered by standard Gaussian or Bessel beam illuminations. Moreover, the droplet illumination can be directly combined with the existing methods to achieve rapid image acquisition, such as roller shutter^28^, and further implemented in the nonlinear regime, where an even better energy confinement can be achieved across the central peak, but this at the cost of an increased risk of sample photodamage and a typically lower spatial resolution. Given the improved energy confinement, the droplet illumination can further limit both photobleaching and phototoxicity of the fluorescent molecules that induce sample degradation. As such, our findings pave the way to rapid, high-contrast volume imaging of cells and tissues, heralding novel *in-vivo* biomedical applications.

## Methods

### Sample preparation

Fluorescent beads (Neon Nights) are suspended in ultra pure water at a concentration of 10 *µ*l/ml by vigorous pipetting. Gelatin (Sigma, Type A, 300 bloom) is added to the suspension of beads at a concentration of 40 mg/ml and kept at 37 °C in a water bath until complete solubilisation. The beads/gelatin solution is then transferred in a glass cuvette and kept at 4 °C for 2 h to induce the formation of a physical gel of gelatin.

## Electronic supplementary material

Supplementary Figure 1

Video 1
