# Supplementary material for: Cancellation of Bessel beam side lobes for high-contrast light sheet microscopy
Source: Sci Rep. 2018 Nov 21;8:17178. doi: 10.1038/s41598-018-35006-1 (PMC6249239; doi:10.1038/s41598-018-35006-1)
Supplement: Supplementary file 1 — Supplementary Figure 1 [file 41598_2018_35006_MOESM1_ESM.pdf]

Supplementary information related to manuscript:

"Cancellation of Bessel beam side lobes for high-contrast light sheet microscopy"

Authors:

Giuseppe Di Domenico, Giancarlo Ruocco, Cristina Colosi, Eugenio DelRe, Giuseppe Antonacci

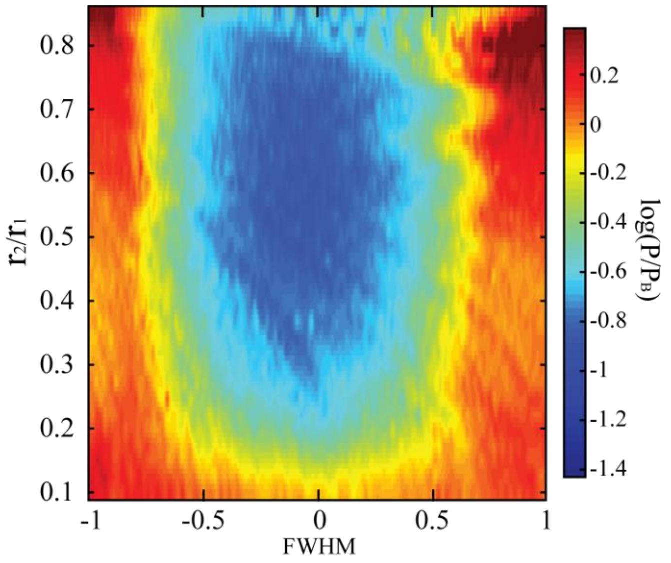

Figure S1 – Map of the experimental side lobes power P integrated for a full axial period (Δz=2*FWHM) of the droplet beam with respect to the side lobe power P_B of a Bessel beam. At the droplet peak center, the side lobe power is overall significantly lower than the Bessel beam whilst it starts to increase towards the minima of the droplet peak.
